# Synthesis of silver sulfide nanoparticles and their photodetector applications†

**DOI:** 10.1039/c8ra03306d

**Published:** 2018-08-09

**Authors:** Myung Hyun Kang, Sung Ho Kim, Seunghun Jang, Ji Eun Lim, Hyunju Chang, Ki-jeong Kong, Sung Myung, Joung Kyu Park

**Affiliations:** Advanced Materials Division, Korea Research Institute of Chemical Technology Daejeon Korea parkjk@krict.re.kr; Center for Molecular Modeling and Simulation, Korea Research Institute of Chemical Technology Daejeon Korea

## Abstract

Silver sulfide nanoparticles (Ag_2_S NPs) are currently being explored as infrared active nanomaterials that can provide environmentally stable alternatives to heavy metals such as lead. In this paper, we describe the novel synthesis of Ag_2_S NPs by using a sonochemistry method and the fabrication of photodetector devices through the integration of Ag_2_S NPs atop a graphene sheet. We have also synthesized Li-doped Ag_2_S NPs that exhibited a significantly enhanced photodetector sensitivity *via* their enhanced absorption ability in the UV-NIR region. First-principles calculations based on a density functional theory formalism indicated that Li-doping produced a dramatic enhancement of NIR photoluminescence of the Ag_2_S NPs. Finally, high-performance photodetectors based on CVD graphene and Ag_2_S NPs were demonstrated and investigated; the hybrid photodetectors based on Ag_2_S NPs and Li-doped Ag_2_S NPs exhibited a photoresponse of 2723.2 and 4146.0 A W^−1^ respectively under a light exposure of 0.89 mW cm^−2^ at 550 nm. Our novel approach represents a promising and effective method for the synthesis of eco-friendly semiconducting NPs for photoelectric devices.

## Abstract 2

Silver sulfide nanoparticles (Ag_2_S NPs) are currently being explored as infrared active nanomaterials that can provide environmentally stable alternatives to heavy metals such as lead.

## Graphical abstract

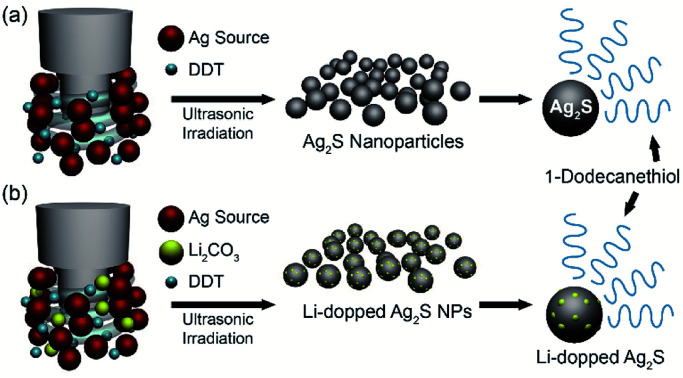

## Introduction

Ultraviolet (UV), visible, and near infrared (NIR) imaging with nanoparticles (NPs) has been extensively investigated owing to their utility in many applications, such as electroluminescent devices, optical modulators, photodetectors, and biological fluorophores.^1–9^ Recently, quantum dot NPs, such as PbS and PbSe, have particularly emerged as some of the most promising new materials for the development of advanced photoelectric devices, because of low-cost manufacturing, solution processability, and their superior photoelectric properties including unique absorption and emission of light in the NIR regions.^10–15^ However, lead-containing NPs are highly toxic and responsible for extensive environmental contamination and health problems in various parts of the world.^16,17^ In order to overcome these limitations, the Ag_2_S NPs have been extensively studied as attractive NIR-absorbing NPs for photovoltaics, photoconductors, and IR detectors due to their high biocompatibility and unique absorption ability in the UV-NIR regions, which can be used as multispectral photodetectors in UV, visible, and NIR spectrum because of their light absorption in a broad wavelength range.^18–27^ However, the development of a facile and easy methods for the preparation of the high-quality and monodisperse Ag_2_S NPs is still required for routine industrial applications including photoelectric devices and photodetectors.

In this study, we demonstrate the facile preparation of Ag_2_S NPs and one-pot synthesis of Li-doped Ag_2_S NPs *via* ultrasonic irradiation, which resulted in a dramatic enhancement of their absorption and emission capabilities in the NIR region. The effect of Li ion doping on the electronic structure of the Ag_2_S system was also investigated by first-principles calculations, which indicated that the Li-doped Ag_2_S NPs could enhance the photoluminescence of semiconducting nanocrystals. Finally, hybrid photodetectors based on transparent CVD graphene nanosheets and Ag_2_S NPs were successfully fabricated. These photodetectors based on pristine Ag_2_S NPs and Li-doped Ag_2_S NPs showed photoresponses of 2723.2 and 4146.0 A W^−1^, respectively, under a light exposure of 0.89 mW cm^−2^ at 550 nm. The proposed synthesis and doping methods constitute a facile and efficient approach for fabricating graphene-NP-based hybrid two-dimensional (2D) photoelectric devices for various applications, including flexible devices and advanced photo-transistors.

## Results and discussion

**Fig. 1:**
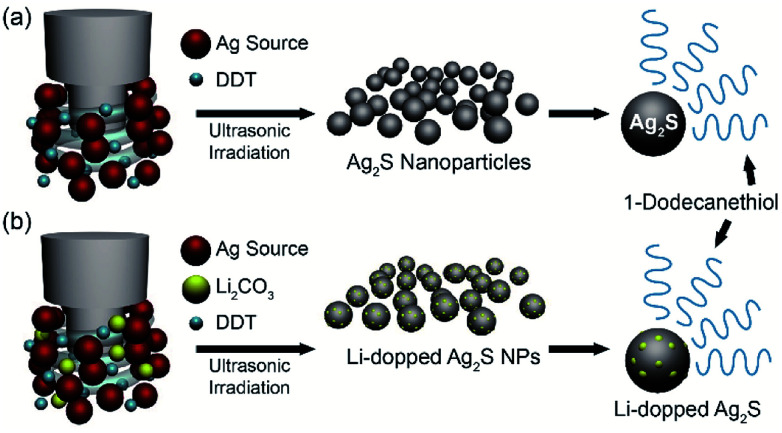
Synthesis of Ag_2_S NPs and Li-doped Ag_2_S NPs. Schematic illustration of the synthesis of (a) Ag_2_S NPs and (b) Li-doped Ag_2_S NPs by using ultrasonic irradiation.

The facile synthesis of 10 nm Ag_2_S NPs was performed by using a sonochemical method, in which the decomposition of raw materials is induced by ultrasound under ambient conditions (Fig. 1a). Silver nitrate (AgNO_3_) in 1-dodecanethiol was sonicated to generate localized hot spots within the acoustic cavitation of collapsing bubbles during ultrasonic irradiation (reaction time: 10 min, power: 50%, temperature: ∼160 °C) (see Fig. S1 in ESI †). In order to improve NIR photodetector sensitivity, Li-doped Ag_2_S NPs were also synthesized using a method similar to that used for pristine Ag_2_S NPs by adding the appropriate amount of Li in the reaction bottle (Fig. 1b). Such photosensitive materials can lead to an enhancement of the absorption ability in the broad wavelength range, resulting in an overall improvement of their photodetector performance.

**Fig. 2:**
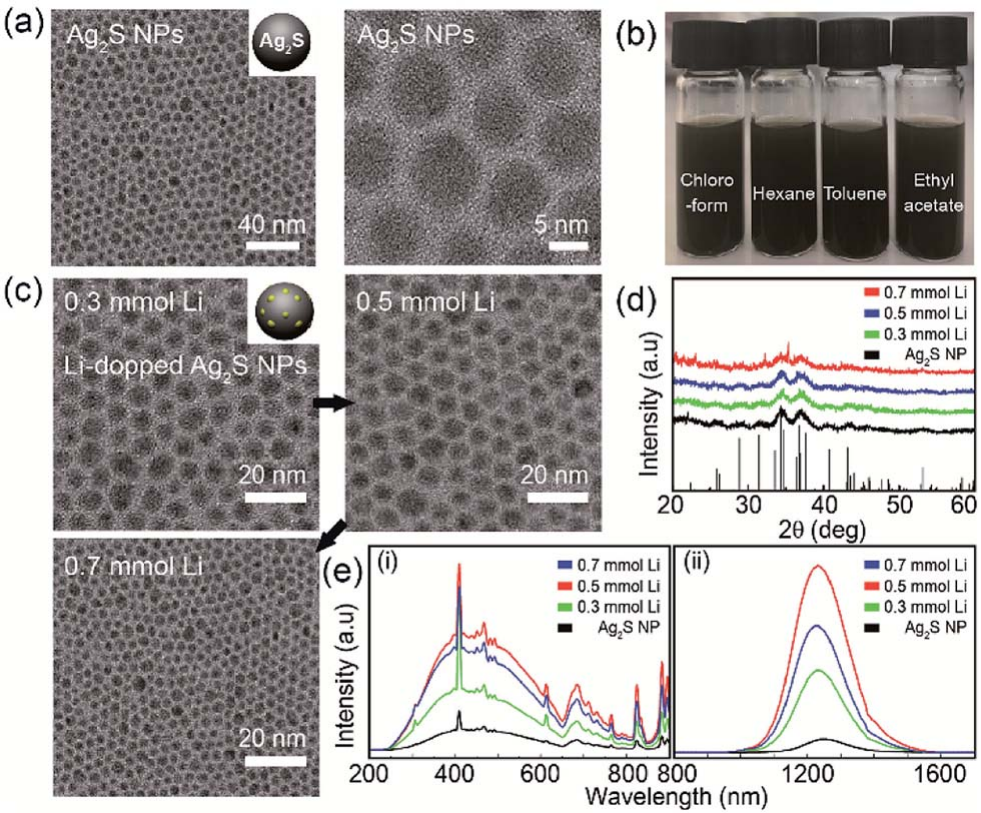
Synthesis of Ag_2_S NPs and Li-doped Ag_2_S NPs. (a) Representative TEM images of Ag_2_S NPs with a 10 nm size. (b) Optical images of the dispersion of Ag_2_S NPs in chloroform, hexane, toluene, and ethyl acetate. (c) XRD patterns of Ag_2_S NPs in terms of Li concentrations. (d) TEM images for 0.3 mmol Li-doped (right), 0.5 mmol Li doped (middle), and 0.7 mmol Li-doped (left) Ag_2_S NPs. (e) UV-Vis-NIR absorption spectra (left) and PL emission spectra under an excitation of 850 nm (right).

Transmission electron microscopy (TEM) analysis was used to characterize the structure and morphology of Ag_2_S NPs and Li-doped Ag_2_S NPs. The TEM images of the as-prepared Ag_2_S NPs confirmed the monodispersity and narrow size distribution of the NPs (Fig. 2a) that can be attributed to the effective separation of the nucleation and growth processes during ultrasonic irradiation at 160 °C. This result was similar to that reported in a previous study.^28^

Significantly, the Ag_2_S NPs were well dispersed in various organic solvents due to the presence of dodecanethiol coated on the NP surface (Fig. 2b). In the XRD patterns of the Ag_2_S NPs doped with different amounts of Li^+^, most of the peaks corresponded to the monoclinic Ag_2_S phase (JCPDS no. 014-0072) (Fig. 2d). The TEM images of Li-doped Ag_2_S NPs for different Li^+^ amounts revealed spherical monodisperse NPs; Li contents did not affect particle morphology and size (Fig. 2c). The photoluminescence (PL) excitation and emission spectra of Ag_2_S NPs as a function of the Li concentration are shown in Fig. 2e. Unlike general quantum dots, involving PbSe and PbS, these NPs could be effectively excited across the UV-NIR region (See Fig. S2 in ESI†). It has been reported that the Ag_2_S NPs emits efficiently under the various excitation ranges, which makes them promising candidates for photodetectors requiring unique absorption in the various wavelength regions (from UV to NIR). As the content of Li^+^ increased, the emission intensities of the Ag_2_S NPs increased up to 0.05 mmol and exhibited an emission peak at 1250 nm (Fig. 2e). The Li-doped Ag_2_S NPs were found to display a remarkable enhancement of the emission intensity (up to two orders) compared to that of the undoped Ag_2_S NPs. It is well known that even at very small concentrations, Li^+^ ions play an important role as co-dopants in increasing the luminescent efficiency of phosphors.^28^ In the XPS spectrum, only Ag and S peaks are observed and Li 1S peak is unable to distinguish with Ag 4P at around 55 eV because Li 1S peak and Ag 4P peak overlap each other (see Fig. S3 in ESI †). But ICP analysis confirmed the existence of Li in these Ag2S NPs (see Fig. S4 in ESI †).

**Fig. 3:**
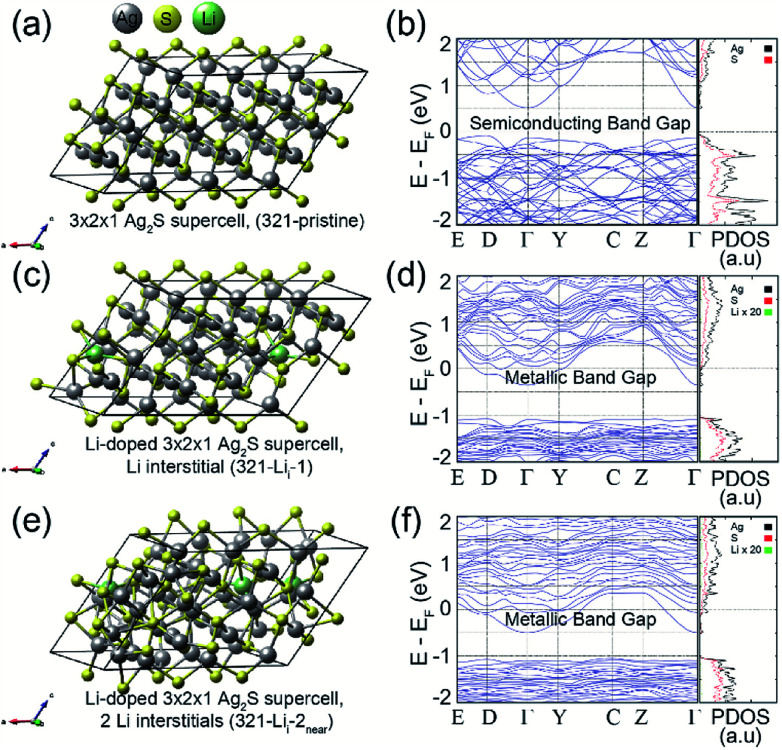
Calculations for the pristine and Li doped (interstitials) 3 × 2 × 1 Ag_2_S. Calculated optimized atomic geometries for the (a) 321-pristine, (c) 321-Li_i_-1, and (e) 321-Li_i_-2_near_. The gray, yellow, and green balls represent the Ag, S, and Li atoms, respectively. Calculated band structures and PDOSs for (b) 321-pristine, (d) 321-Li_i_-1, and (f) 321-Li_i_-2_near_. The Fermi levels of all calculated systems were set at zero.

To understand the effect of Li ion doping on the electronic structures of the Ag_2_S system, we performed first-principles calculations based on the density functional theory formalism for the Li interstitials and substitutions in crystalline Ag_2_S. Fig. 3 (left) shows the optimized atomic geometries of (a) 3 × 2 × 1 Ag_2_S supercell (321-pristine), (c) 3 × 2 × 1 Ag_2_S supercell with a Li interstitial (321-Li_i_-1), and (e) 3 × 2 × 1 Ag_2_S supercell with two Li interstitials (321-Li_i_-2_near_). As shown in Fig. 2d, since the Li contents did not affect the structural properties of Ag_2_S NPs, not only interstitial doping but also substitution were considered in our calculations. The Li concentration was about 0.12 wt% for the case of 321-Li_i_-1, which corresponded to the experimental conditions. Although a Li interstitial was added in the interspace of the Ag_2_S cell, its crystal structure was well preserved, as shown in Fig. 3c. Furthermore, when an additional Li atom was appended, a local amorphous structure in the doped Ag_2_S began to appear (Fig. 3e). Additionally, the calculations for the Li substitutions at the Ag sites in the 3 × 2 × 1 A Ag_2_S supercell were also carried out (See Fig. S5 in ESI†).

Since Li^+^ ions (0.9 Å) have a smaller ionic radius than Ag^+^ ions (1.3 Å) in the Ag_2_S crystal, regardless of the number (per unit-cell) of Li substitutions, the crystal structures of doped Ag_2_S systems with Li substitutions at the Ag sites were well maintained.^29^ Likewise, the electronic structure of Ag_2_S hardly changed upon Li substitution (See Fig. S5 in ESI†). However, the Li interstitial in the Ag_2_S crystal caused a significant change in the electronic structure. Fig. 3 (right) shows the calculated band structures and projected density of states (PDOS) of (b) 321-pristine, (d) 321-Li_i_-1, and (f) 321-Li_i_-2_near_. As shown in the band structures of Fig. 3b and d, the bottom of the conduction band shifted below the Fermi level by the Li interstitial doping. As a result, 321-Li_i_-1 exhibited a metallic band gap. The Li impurity provides a partial 2s electron to the pristine Ag_2_S system, which leads the change in band structure from a semiconducting to a metallic band gap.^30^

In the case of the 321-Li_i_-2_near_ system with two Li interstitials, it could be confirmed that the bottom of the conduction band was reduced to a lower energy level than that of 321-Li_i_-1 by an additional Li 2 s partial electron (Fig. 3d and f). In order to check the effect of the Li–Li interaction on the electronic structure of the Li doped-Ag_2_S system, the calculations for the 3 × 2 × 1 Ag_2_S supercells with two Li interstitials (or two Li substitutions) were performed by varying the Li–Li interdistance (See Fig. S6 or S5c–f in ESI†). However, no noticeable influence of the electronic structure on the interaction between two Li interstitials (or two Li substitutions) in the Ag_2_S system was observed.

**Fig. 4:**
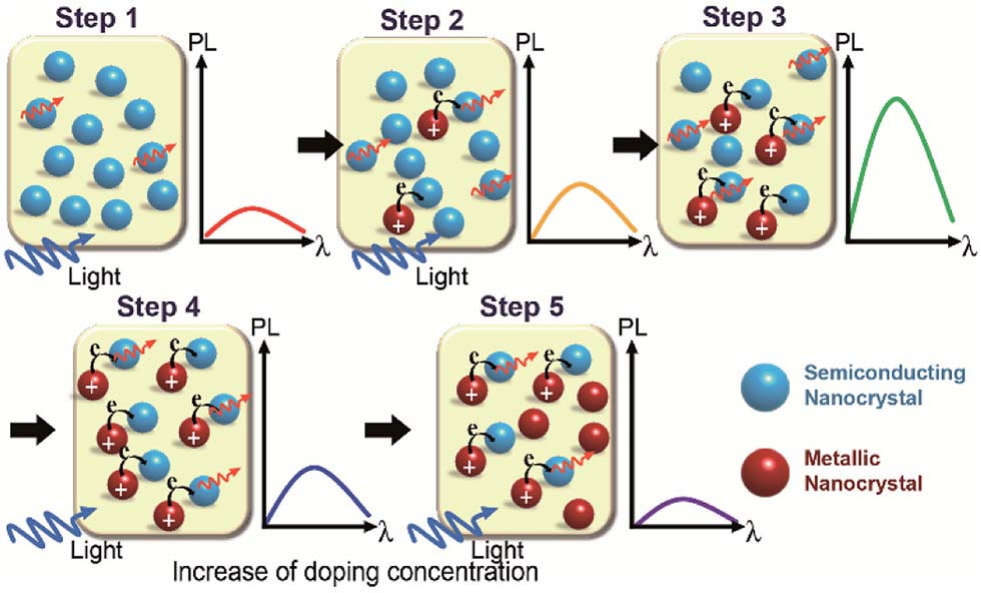
Mechanism of photoluminescence enhancement and quenching Li-doped Ag_2_S NPs. Schematic diagram for the mechanism of the photoluminescence enhancement and quenching in Li-doped Ag_2_S NPs by increasing Li doping concentration (from Step 1 to Step 5, along the black arrow direction). The blue-sky and red balls represent the semiconducting and metallic Ag_2_S NP. The blue and red wave arrows indicate the photoluminescence excitation source and light emission, respectively.

Based on the results obtained from our calculations, we proposed the following mechanism for the photoluminescence enhancement and quenching in Fig. 2e, as shown in Fig. 4. Extra carriers in Li interstitial doped Ag_2_S are accumulated within the crystal, which further enhances its metallic property. By increasing the Li doping concentration, extra electrons in metallic NPs begin to migrate to original semiconducting NP outside (Step 2 in Fig. 4), thus positively charged metallic NPs can enhance the photoluminescence of the semiconducting NPs.

However, this phenomenon takes place only up to optimal doping conditions (Step 3 in Fig. 4). Above a certain doping concentration, the absolute number of semiconducting NPs participating in the photoluminescence process are reduced compared to that of metallic NPs, and thereby the total luminescence of the sample begins to decrease rather than increase (from Step 4 to Step 5 in Fig. 4). A similar mechanism of photoluminescence enhancement and quenching has been already proposed in a previous study on Ag-doped CdSe NP.^31^

**Fig. 5:**
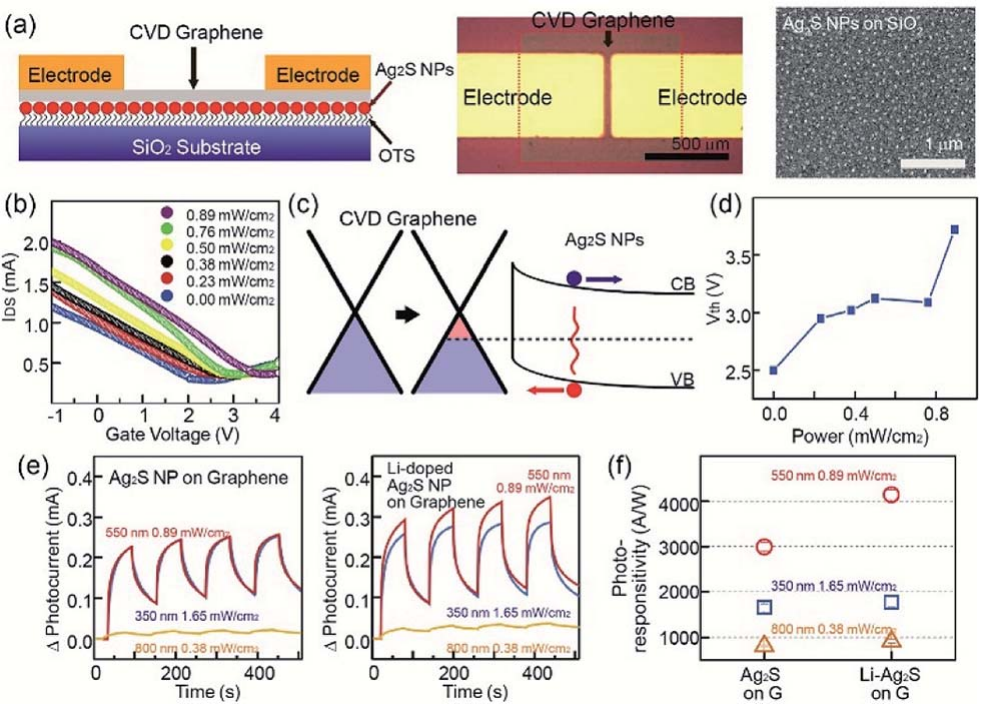
Photodetectors based on graphene and Ag_2_S NPs (a) a schematic diagram of the structure of a photodetector based on graphene and Ag_2_S NPs (left). Optical image of the photodetectors (middle). SEM image of graphene on Ag_2_S NPs (right). (b) The transfer characteristics of a transistor based on graphene and Ag_2_S NPs under various light exposing conditions. (c) A schematic energy band diagram of the Fermi level variation of graphene and Ag_2_S NPs. (d) Threshold voltage of the devices as a function of light power. (e) Photocurrent of photodetector based graphene-Ag_2_S NPs (left) and graphene Li-doped Ag_2_S NPs (right) under cyclic light exposure. Here, a light source of 0.89 W m^−2^ at 550 nm, 1.65 W m^−2^ at 350 nm, and 0.38 W m^−2^ at 800 nm was used for the measurements. (f) Photoresponsitivity of photodetectors based graphene-Ag_2_S NPs and graphene Li-doped Ag_2_S NPs obtained by light exposure of 0.89 W m^−2^ at 550 nm, 1.65 W m^−2^ at 350 nm, and 0.38 W m^−2^ at 800 nm.

In addition, Ag_2_S NPs and Li-doped Ag_2_S NPs were successfully employed for the fabrication of hybrid photodetectors based on CVD graphene and photosensitive NPs. Recently, carbon-based materials, such as carbon nanotubes, graphene oxide, and graphene have drawn considerable attention due to their superior optical, electrical, and mechanical properties.^32^ Among carbon materials, graphene and graphene-related materials have been proposed as strong candidates for various optoelectronic applications, such as ultra-broadband photodetectors and solar cells.^33^ However, their low optical absorption and the short recombination rate have limited their application to graphene-based optoelectronic devices. First, 1-octadecyltrichlorosilane (OTS) was utilized as self-assembled monolayer (SAM) molecules to achieve the uniform assembly of Ag_2_S NPs. In this case, a UV/ozone treatment of the SiO_2_ surface was carried out, and the UV-treated SiO_2_ substrate was placed in the OTS solution to cover the SiO_2_ surface. When the OTS-coated SiO_2_ substrate was placed in the solution of methyl-terminated Ag_2_S NPs, the Ag_2_S NPs were assembled on the neutral-charged OTS molecular layer. CVD-grown graphene nanosheets were transferred on the top of Ag_2_S NPs using a PMMA-assisted wet transfer method. Finally, Au/Cr acting as source and drain electrodes were deposited using a shadow mask, while 1-butyl-3-methylimidazolium (BmimPF_6_) was used as the ionic liquid. Ag_2_S NPs were used as photosensitive materials. As shown in the image in Fig. 5a, the channel length and width of the device were 100 μm and 500 μm, respectively. The SEM image in Fig. 5a and atomic force microscopy (AFM) image showed that the NP density on the SiO_2_ substrate was uniform (See Fig. S8 in ESI†), and the NP density was approximately 150 μm^−2^. The transfer curve (*I*_DS_–*V*_G_) of pristine graphene devices at *V*_DS_ = 0.1 V exhibited a charge-neutral Dirac point at a near-zero gate voltage (*V*_G_) and an asymmetric hole and electron conduction (see Fig. S9 in ESI†). In the case of devices based on Ag_2_S NPs and graphene, the Dirac voltage showed a positive shift compared with that of pristine graphene devices, and this positive shift was increased with increase of the illumination power. The electrical band structures provide a good explanation of the difference between the photodetectors based on graphene and Ag_2_S NPs, as shown in Fig. 5c. The energy-band bending at the interface between a surface of graphene and Ag_2_S NPs also occurred. The direction of the built-in electric field was formed from the graphene to the Ag_2_S NPs. The photo-generated electrons (or holes) in the Ag_2_S NPs moved within the graphene sheet with the passage of the energy band bending at the interface under the light illumination.^34–36^ The photodetector showed an increase of current level under the exposure to a light source. Here, holes transfer from Ag_2_S NPs to graphene resulted in an increase in the photocurrent (Fig. 5c). Fig. 5d shows the Ag_2_S-graphene photodetector under a light exposure of 0.00, 0.23, 0.38, 0.50, 0.76, and 0.89 mW cm^−2^ exhibited a Dirac voltage of 2.2, 2.4, 2.6, 2.9, 3.0 and 3.5 V, respectively.

The photoresponse characteristics of hybrid photodetectors were evaluated by measuring the time-dependent photocurrent under various illumination conditions, such as 0.89 W m^−2^ at 550 nm, 1.65 W m^−2^ at 350 nm, and 0.38 W m^−2^ at 800 nm. First, in the case of photodevices based on pristine Ag_2_S NPs, the current through the graphene channel increased under light exposure. Moreover, we observed that the photocurrent under the light exposure at 350- and 550 nm wavelengths was larger than that under 800 nm, as shown in Fig. 5e. In addition, as previously mentioned, Li-doped Ag_2_S NPs possess superior light absorption properties than pristine Ag_2_S NPs, while photodetectors based on graphene and Ag_2_S NPs exhibit a larger photocurrent than Ag_2_S NPs. Fig. 5f shows the photoresponse of our devices under the various exposing conditions. For example, hybrid photodetectors based on Ag_2_S NPs and Li-doped Ag_2_S NPs displayed a photoresponse of 2723.2 and 4146.0 A W^−1^, respectively, under a light exposure of 0.89 mW cm^−2^ at 550 nm. In order to investigate the stability of the NPs, the photo and thermal stability of NPs was studied by illuminating the NPs using UV (365 nm) and heat-treated temperatures for various times. The PL emission intensities of the samples does not show much reduction in PL emission intensities, which shows the NPs have high photo and thermal stability. Moreover, to investigate the long-term air-stability, graphene photodetectors with based a graphene transistor with Ag_2_S NPs and Li-doped Ag_2_S NPs were placed in an environmental chamber, with a relative humidity and temperature of 50% and 25 °C, respectively. Our photodetectors exhibited a similar trend with NPs data for 90 days, possibly due to charge trapping from water molecules on the graphene surface, which shows high reliability and stability of the NPs as well as photodetector devices (see Fig. S10 and S11 in ESI†).

## Experimental

### Materials

Silver nitrate (AgNO_3_, 99%), Li_2_CO_3_ (98%), and 1-dodecanethiol (DDT) were purchased from Sigma Aldrich. Chloroform and ethyl acetate were used to disperse and to isolate the NPs. All chemicals were used without further purification.

### Synthesis of Ag_2_S NPs and Li-doped Ag_2_S NPs

The Ag_2_S and Li-doped Ag_2_S NPs were synthesized by ultrasonication. For the synthesis of Ag_2_S NPs, AgNO_3_ was added to a 20 mL vial containing 10 mL of DDT. The solution was treated by ultrasound irradiation for 10 min in air atmosphere. The resulting suspension was centrifuged with ethyl acetate several times to remove any by-products and dried in an electric oven at 80 °C. The Li-doped Ag_2_S NPs were synthesized in the same way, upon addition of the appropriate amount of lithium to the reaction bottle.

### Characterization of Ag_2_S and Li-doped Ag_2_S NPs

The absorption spectra of solutions containing 0.01 g of Ag_2_S and Li-doped Ag_2_S NPs (in 10 mL of chloroform) were measured using a SolidSpec-3700 UV-Vis-NIR spectrophotometer from Shimadzu. The photoluminescence (PL) spectra were measured by a Fluorolog3 with TCSPC mode (HORIBA Scientific). All samples were excited by a CW 450 W xenon source, and directed to a single-grating spectrometer. The PL spectra were obtained using a InP/InGaAs detector equipped with an LN cooler. All TEM images were acquired on a JOEL JEM-2100F transmission electron microscope operating at 200 KV. The TEM samples were prepared by drop-casting very thin nanoparticle solutions onto a 200 mesh copper grid with a carbon film (Ted Pella). X-ray diffraction spectroscopy (XRD) for the NPs was performed using a Rigaku D/MAX-220 V X-ray diffractometer equipped with Cu K-alpha (1.540598 Å) source.

### Computational details

The atomic and electronic structures of the pristine and Li-doped Ag_2_S systems were examined using the Vienna *ab initio* simulation package (VASP).^37,38^ The exchange correlation functional was approximated using the PBEsol (Perdew–Burke–Ernzerhof revised for solids) expression.^39^ The electron–ion interactions were modeled using the projector augmented wave (PAW) method.^40^ The electronic wave functions were expanded in a basis set of plane waves using a kinetic energy cutoff of 500 eV. Geometry relaxation steps were performed under the criterion that ionic forces were reduced below 0.02 eV Å^−1^. Doped Ag_2_S systems with interstitial and substitutional Li atoms were analyzed using a unit cell (1 × 1 × 1) and 3 × 2 × 1 supercells with 10 × 5 × 5 and 4 × 3 × 5 *k*-point meshes, respectively. The crystal data of β-Ag_2_S were consistent with earlier reports and were used as the lattice parameters of the pristine Ag_2_S system in this work.^41,42^ The pristine Ag_2_S system possessed a monoclinic crystal structure (space group *P*2_1_/*c*, *a* = 4.231 Å, *b* = 6.930 Å, *c* = 9.526 Å, *β* = 125.48°), as shown in Fig. S7a in ESI.†

### Fabrication of photodetectors based on Ag_2_S (or Li-doped Ag_2_S NPs) and graphene

First, a SiO_2_ surface was subjected to a UV/ozone treatment at atmospheric pressure, and then the UV-treated SiO_2_ substrate was placed in an OTS solution (1/500 by volume ratio in anhydrous hexane). After the OTS-coated SiO_2_ substrate was placed in a 0.16 wt% solution of Ag_2_S NPs and Li-doped Ag_2_S (total solution of 0.1 g NPs under 50 mL CHCl_3_) usually for about 5 minutes, the Ag_2_S NPs were assembled on the OTS molecular layer. CVD-grown graphene nanosheets were transferred on top of the Ag_2_S NPs using a PMMA-assisted wet transfer method. Au/Cr (as source/drain electrodes) were deposited using a shadow mask. In our study, Cr (7 nm)/Au (70 nm), 1-butyl-3-methylimidazolium, and Ag_2_S NPs were used as the source/drain electrodes, ionic liquid, and photosensitive materials, respectively.

## Conclusion

In summary, the facile preparation of Ag_2_S NPs and Li-doped Ag_2_S NPs was successfully achieved for the development of high-performance optoelectronic devices. These materials showed enhancements of absorption and emission in the NIR region through a one-step process upon ultrasonic irradiation. The effect of Li ion doping on the electronic structure of the Ag_2_S was also investigated by first-principles calculations, which indicated that the Li-doped Ag_2_S NPs could have enhanced the NIR photoluminescence and absorption abilities.

Finally, hybrid photodetectors based on transparent CVD graphene nanosheets and Ag_2_S NPs were successfully fabricated. These photodetectors based on pristine Ag_2_S NPs and Li-doped Ag_2_S NPs showed photoresponses of 2723.2 and 4146.0 A W^−1^, respectively, under a light exposure of 0.89 mW cm^−2^ at 550 nm. This approach provides a facile high-quality synthesis method for NPs as well as a doping method for the fabrication of advanced hybrid photoelectric devices such as advanced photo-transistors, and solar cells.

## Conflicts of interest

There are no conflicts to declare.

## Supplementary Material

RA-008-C8RA03306D-s001
