# Supplementary material for: Synthesis of silver sulfide nanoparticles and their photodetector applications
Source: RSC Adv. 2018 Aug 9;8(50):28447–52. doi: 10.1039/c8ra03306d (PMC9083940; doi:10.1039/c8ra03306d)
Supplement: RA-008-C8RA03306D-s001 [file RA-008-C8RA03306D-s001.pdf]

Electronic Supplementary Material (ESI) for RSC Advances.
This journal is © The Royal Society of Chemistry 2018

Supporting Information

Synthesis of silver sulfide nanoparticles and their photodetector applications

Myung Hyun Kang[a], Sung Ho Kim[a], Seunghun Jang[b], Ji Eun Lim[a], Hyunju Chang[b], Ki-Jeong Kong[b], Sung Myung*[a], and Joung Kyu Park*[a]

[a]Advanced Materials Division, Korea Research Institute of Chemical Technology, Daejeon, Korea, E-mail: parkjk@krict.re.kr

[b]Center for Molecular Modeling and Simulation, Korea Research Institute of Chemical Technology, Daejeon, Korea

[#]M. H. Kang, S. H. Kim, and S. Jang contributed equally to this work.

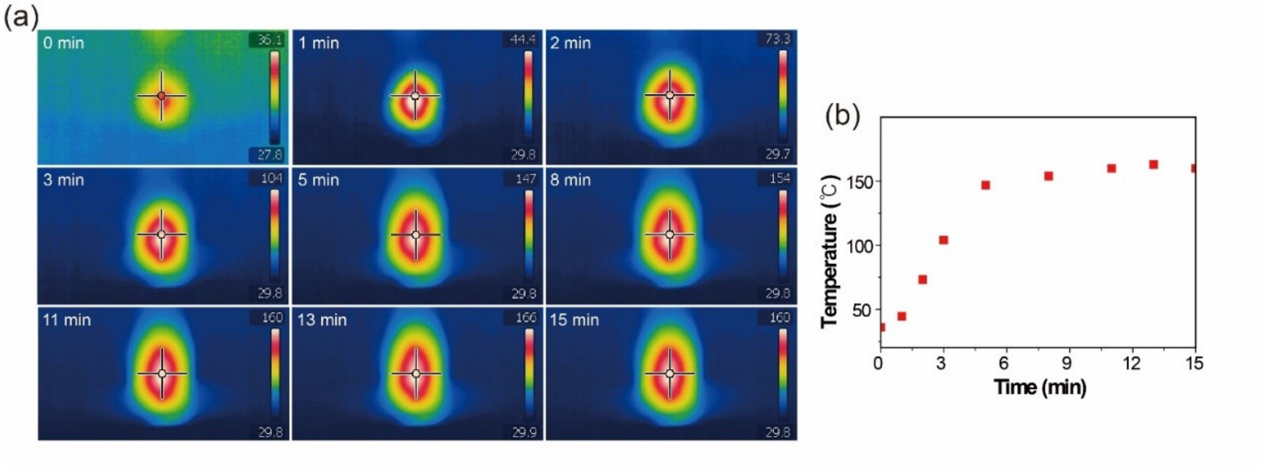

Fig. S1. Sonochemical synthesis of Ag_2S NPs. (a) Thermal images of the reaction vial taken by an infrared camera for the synthesis of Ag_2S NPs. The sonicator in the vial heated the reaction solution up to about 160 °C. The ultrasonic irradiation time usually takes 10 ~ 15 minutes for the synthesis of Ag_2S NPs. (b) The temperatures of the local hot spot were plotted. The temperature may reach 160 °C after 5 minutes of sonication.

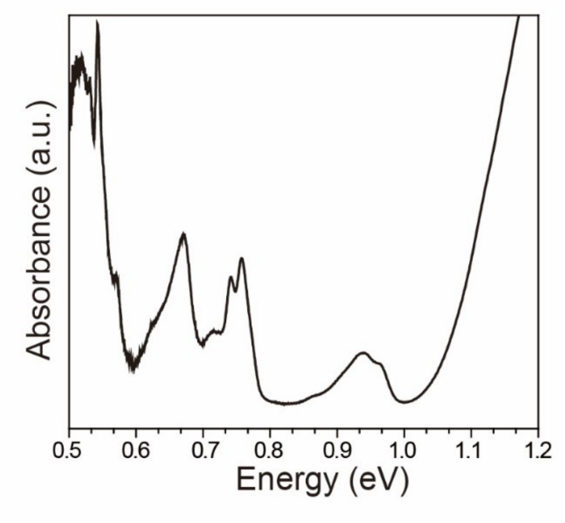

Fig. S2. Absorption spectrum of Ag_2S NPs. 0.01 g of Ag_2S NPs were dispersed in 10 ml Chloroform and measured using a SolidSpec-3700 UV-Vis-NIR spectrophotometer from Shimadzu.

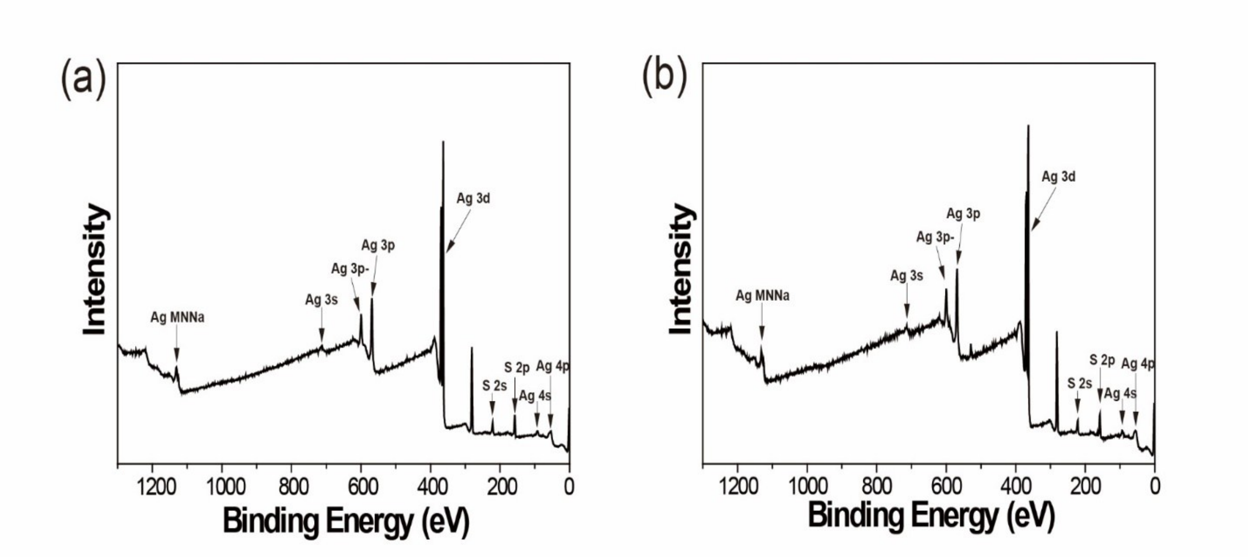

Fig. S3. XPS spectra of (a) Ag_2S and (b) Li-doped Ag_2S NPs.

	Ag (wt%)	Li (wt%)	S (wt%)
**Ag_2S**	77.9	---	11.7
**Li-doped Ag_2S (0.05 mmol)**	72.6	0.447	11.5

Fig. S4. Ag-S-Li ratios of the Ag_2S and Li(0.05mmol)-doped Ag_2S NPs by ICP-AES.

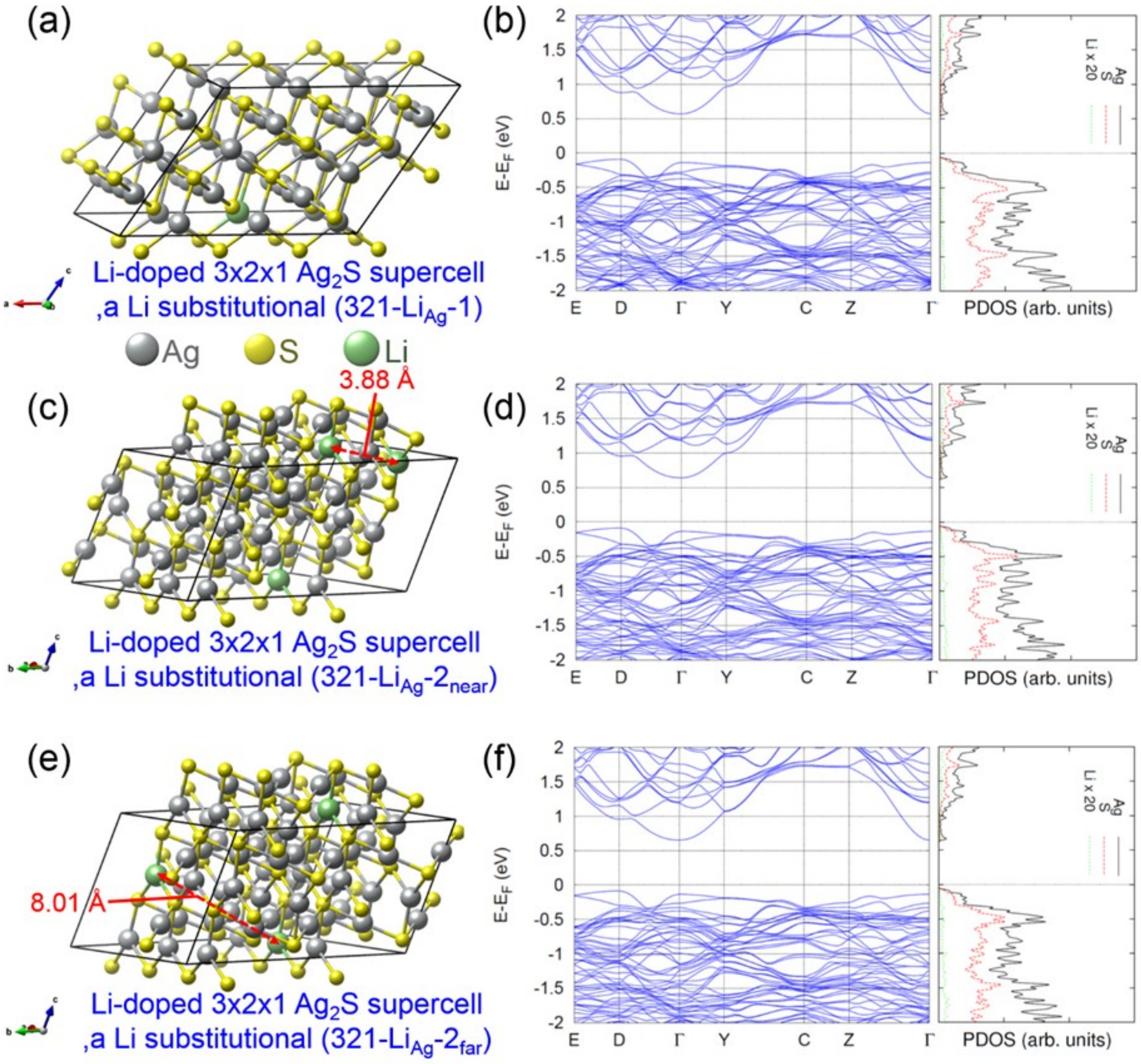

Fig. S5. Calculations for the Li substitutions at the Ag sites in the 3×2×1 Ag_2S supercell. Calculated optimized structures for the (a) 321-Li_{Ag}-1, (c) 321-Li_{Ag}-2_{near}, and (e) 321-Li_{Ag}-2_{far}. The gray, yellow, and green balls represent Ag, S, and Li atoms, respectively. Li-Li distances in 321-Li_{Ag}-2_{near} and 321-Li_{Ag}-2_{far} are 3.88 Å and 8.01 Å. Calculated band structures and PDOSs for the (b) 321-Li_{Ag}-1, (d) 321-Li_{Ag}-2_{near}, and (f) 321-Li_{Ag}-2_{far}. The Fermi levels of all calculated systems were set to zero.

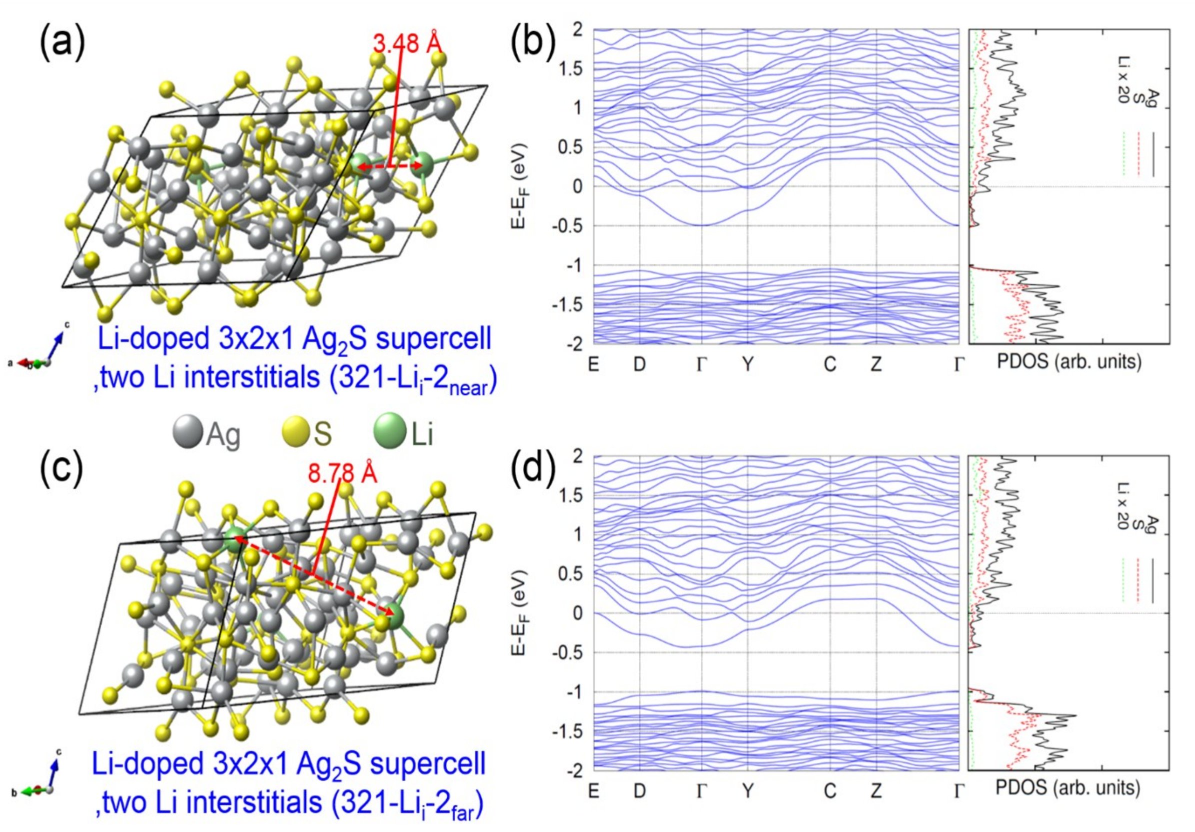

Fig. S6. Calculations for the Li interstitials in the 3×2×1 Ag_2S supercell. Calculated optimized structures for the (a) 321-Li_i-2_{near}, and (c) 321-Li_i-2_{far}. The gray, yellow, and green balls represent Ag, S, and Li atoms, respectively. Li-Li distances in 321-Li_i-2_{near}, and 321-Li_i-2_{far} are 3.48 Å and 8.78 Å. Calculated band structures and PDOSs for the (b) 321-Li_i-2_{near}, and (d) 321-Li_i-2_{far}. The Fermi levels of all calculated systems were set to zero.

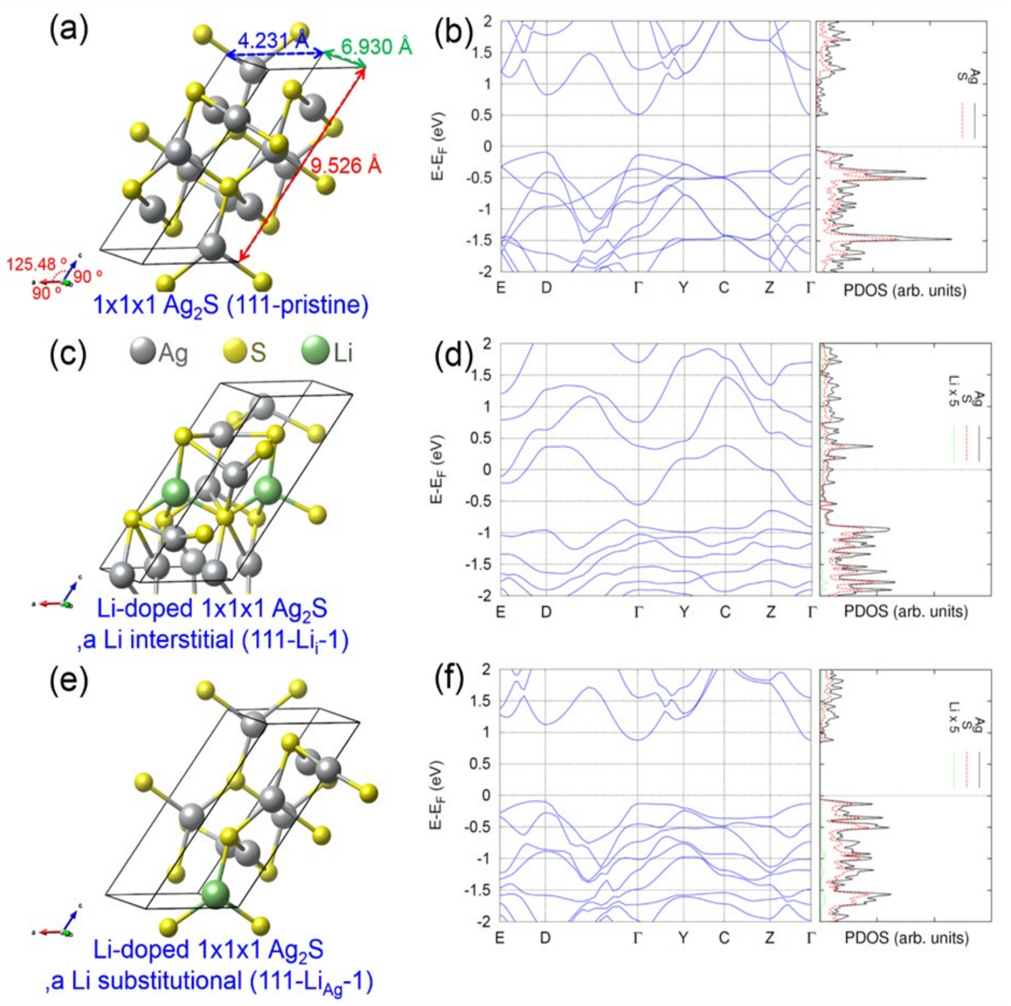

Fig. S7. Calculations for the pristine and Li doped 1×1×1 Ag_2S. Calculated optimized structures for the (a) 111-pristine, (c) 111-Li_i-1, and (e) 111-Li_{Ag}-1. The gray, yellow, and green balls represent Ag, S, and Li atoms, respectively. Calculated band structures and PDOSs for the (b) 111-pristine, (d) 111-Li_i-1, and (f) 111-Li_{Ag}-1. The Fermi levels of all calculated systems were set to zero.

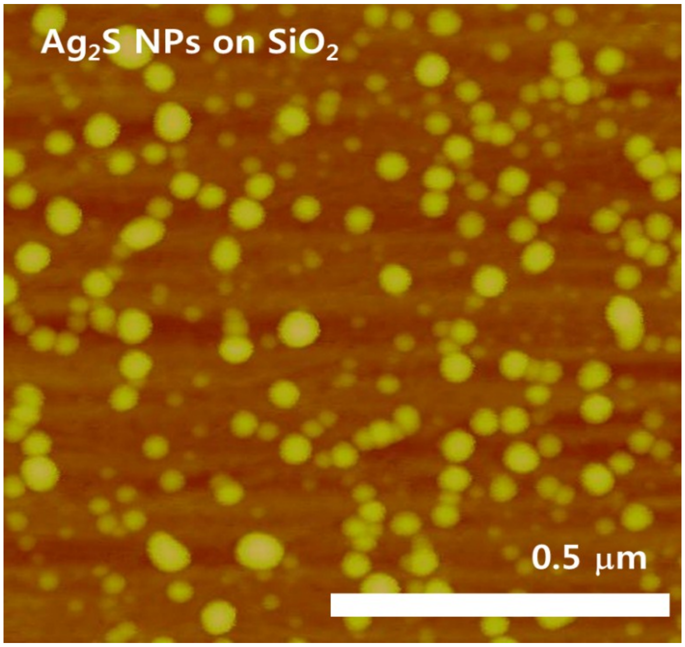

Fig. S8. Atomic force microscopy (AFM) image of Ag_2S NPs density on the SiO_2 substrate.

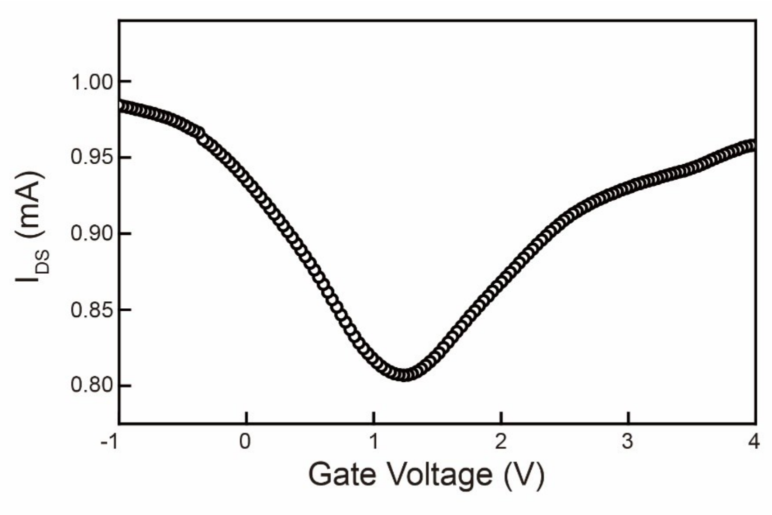

Fig. S9. The transfer curve (I_{DS}-V_G) of pristine graphene devices at V_{DS} = 0.1 V.

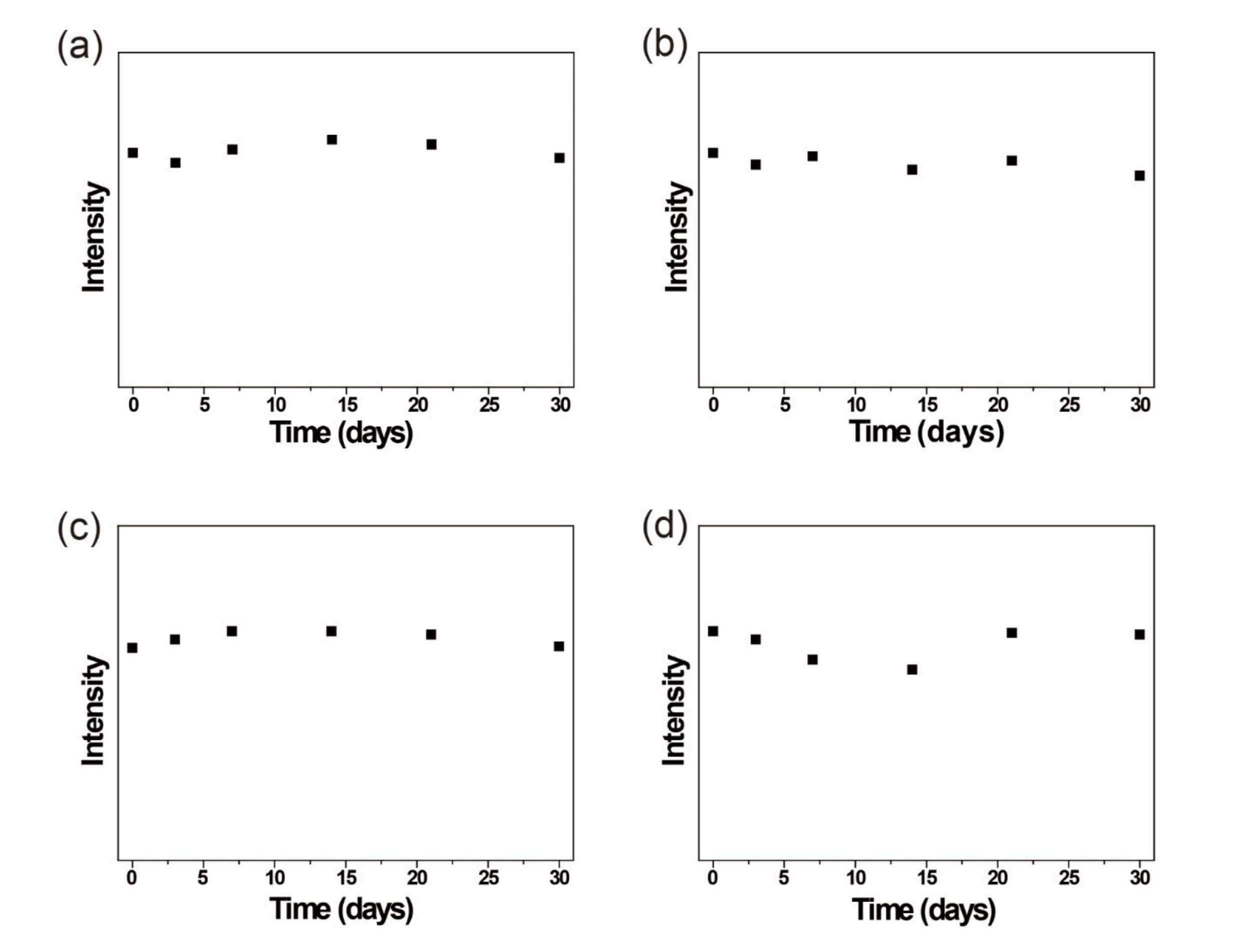

Fig. S10. PL emission intensities of Li-doped Ag_2S NPs (a) UV (365 nm) irradiated for 30 days, (b) RT after 30 days, (c) heat-treated to 70 ℃ for 30 days, and (d) 150 ℃ for 30 days.

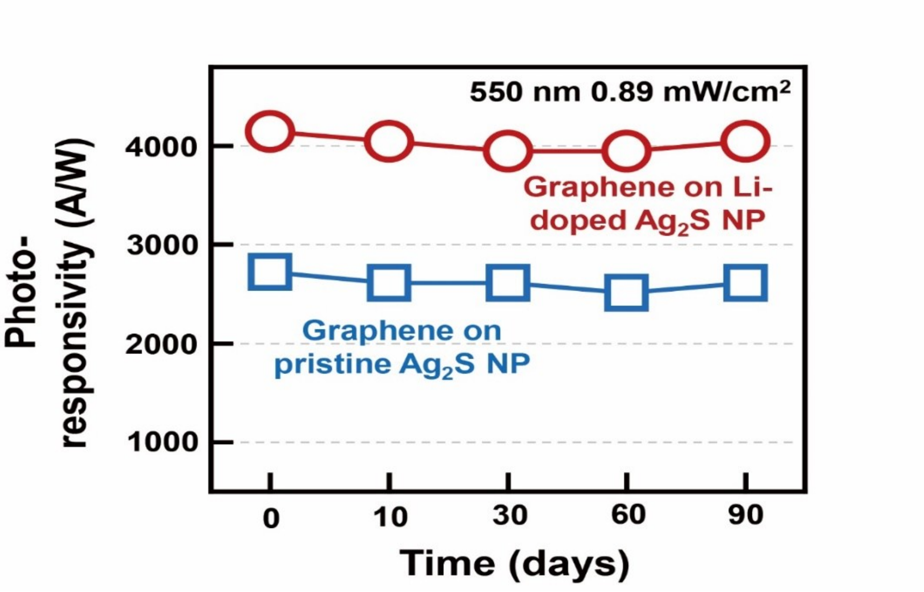

Fig. S11. Long term air-stability test of photodetectors based on graphene and Ag_2S NPs and Li-doped Ag_2S NPs.
